# Identification of a capsid-derived Zika virus epitope with high IgG discriminatory performance

**DOI:** 10.1007/s00705-026-06677-3

**Published:** 2026-06-12

**Authors:** Ana Luisa Cauvila dos Santos, Sandro Rodrigues Lopes, Raíne Piva Amaral, Luiz Cosme Cotta Malaquias, Alice dos Santos Nunes Ferreira, Danilo Bretas de Oliveira, Luiz Felipe Leomil Coelho

**Affiliations:** 1 Laboratório de Vacinas, Departamento de Microbiologia e Imunologia, Universidade Federal de Alfenas, Alfenas, Brazil; 2 Laboratório de Doenças Infecciosas e Parasitárias, Universidade Federal dos Vales do Jequitinhonha e Mucuri, Diamantina, Minas Gerais 39100-000 Brazil

**Keywords:** Elisa, Zika virus, Capsid, Peptide

## Abstract

The identification of specific serological targets for *Orthoflavivirus zikaense* (ZIKV) remains challenging due to the high degree of structural similarity and antibody cross-reactivity among co-circulating arboviruses, particularly dengue virus (DENV). In this context, immunoinformatics-based approaches may contribute to the rational selection of candidate epitopes with potentially improved analytical specificity. In the present study, five conserved ZIKV peptides previously identified through an immunoinformatic pipeline were experimentally evaluated as candidate serological targets. Peptide reactivity was assessed by indirect ELISA using human serum samples positive for anti-ZIKV IgM and IgG antibodies. Distinct reactivity patterns were observed between antibody classes. IgM assays showed limited analytical discriminatory capacity, particularly due to reduced specificity for some peptides. In contrast, IgG-based analyses revealed more favorable analytical profiles. Among the peptides evaluated, a peptide identified in the capsid (PEP03) exhibited the most favorable analytical performance, with an area under the ROC curve (AUC) of 0.9183 (95% CI: 0.8301–1.000). An absence of detectable reactivity was observed among sera from DENV-positive patients, and minimal reactivity with chikungunya virus (CHIKV) positive samples under the experimental conditions employed. Other peptides tested in this study demonstrated variable analytical profiles, reinforcing the importance of rational epitope selection for serological applications. Overall, these findings provide preliminary analytical evidence that structurally derived ZIKV epitopes may exhibit distinct IgM and IgG recognition patterns and support the continued evaluation of capsid-derived epitopes as candidate targets for serological applications (IgG detection) in regions where multiple arboviruses co-circulate.

**Supplementary Information:**

The online version contains supplementary material available at 10.1007/s00705-026-06677-3.

## Introduction

*Orthoflavivirus zikaense*, commonly known as Zika virus (ZIKV), is an arbovirus belonging to the genus *Orthoflavivirus* and the family *Flaviviridae* [1, 2] and has been responsible for large epidemics in different regions of the world [3–10]. More recently, the ZIKV outbreak in the Americas raised significant public health concerns due to its association with neurological complications and congenital anomalies [11, 12]. Although most ZIKV infections are asymptomatic and self-limited, accurate laboratory confirmation is essential due to the potential risk of congenital abnormalities [13, 14]. Molecular diagnostic methods, such as reverse transcription polymerase chain reaction (RT-PCR), are considered the gold standard for ZIKV diagnosis and are recommended by the World Health Organization (WHO). However, their applicability is limited by the short window of viral RNA detection in body fluids and by the requirement for specialized infrastructure and trained personnel [15, 16]. In this context, serological tests represent an important complementary diagnostic approach, particularly for epidemiological surveillance and large-scale applications [17]. Nevertheless, the development of highly accurate serological assays for ZIKV remains challenging due to extensive antigenic similarity and cross-reactivity with other co-circulating arboviruses, especially *Orthoflavivirus denguei* (DENV) [18, 19].

Commercial assays are generally based on recombinant envelope (E) and non-structural protein 1 (NS1) antigens. Although manufacturers report high specificity and sensitivity, independent performance evaluations have demonstrated specificity and sensitivity below expected standards, thereby limiting their discriminatory capacity in endemic regions [20–24]. Although E and NS1 proteins are immunodominant and widely used as antigenic determinants, reliance on these targets may limit the exploration of alternative regions with greater potential for antigenic discrimination. These limitations highlight the need for rational and optimized strategies for the development of alternative antigens with desirable immunological characteristics. In this context, immunoinformatics has emerged as a promising tool for systematic epitope prediction, enabling the identification of conserved and virus-specific immunogenic regions with reduced sequence similarity compared to closely related pathogens. Reverse vaccinology based approaches allow the in silico prediction of epitopes based on antigenicity, structural accessibility, and immunogenicity, significantly accelerating the development of novel immunological targets [25, 26]. However, experimental validation of these candidates remains essential to evaluate their performance and recognition profile by antibodies derived from natural infection.

ZIKV structural proteins represent relevant antigenic sources, as they are directly exposed to the host immune system and participate in the induction of humoral responses. In previous studies conducted by our group [27], a set of epitopes highly conserved among circulating ZIKV strains and showing reduced sequence similarity to other Orthoflavivirus species was identified through an immunoinformatics-based pipeline. In addition to sequence conservation, the selection of candidate epitopes included other parameters such as predicted antigenicity, structural accessibility, and reduced homology with related flaviviruses [27]. All these parameters were used to prioritize peptides with potentially favorable analytical specificity for subsequent experimental evaluation. However, the biological relevance and class-specific antibody recognition of these candidates must be established through experimental evaluation. A crucial aspect of the serological evaluation of antigens is the distinction between IgM and IgG recognition profiles. IgM antibodies are typically associated with early infection and exhibit lower affinity and greater cross-reactivity among flaviviruses [28, 29]. IgG antibodies undergo affinity maturation and may provide improved discriminatory capacity. Therefore, analytical characterization of the class-specific reactivity represents an essential step to determine the translational potential of candidate epitopes identified through immunoinformatics.

In the present study, we experimentally validated five synthetic peptides derived from structural proteins of *Orthoflavivirus zikaense* discovered through an immunoinformatics-guided pipeline. The IgM and IgG seroreactivity profiles were determined using an indirect enzyme-linked immunosorbent assay (ELISA). By integrating computational approaches and experimental evaluation of underexplored ZIKV epitopes, this work seeks to advance rational antigen discovery strategies and clarify the potential relevance of these regions as serological targets in flavivirus-endemic settings.

## Methods

### Peptide synthesis and serum samples

The peptides used in the present study (Table 1) were synthesized based on amino acid sequences identified through previous bioinformatic analyses, according to Salvador et al. (2019). They were synthesized by GenScript (USA) and subsequently solubilized according to their hydrophobicity profiles. After solubilization, the solutions were stored at − 20 °C until use. Human serum samples positive for ZIKV used in this study were kindly provided by the Vaccine Technology Center of the Federal University of Minas Gerais (UFMG) or Federal University of the Jequitinhonha and Mucuri Valleys and and ZIKV infection status was confirmed by PCR and/or serological diagnostic assays (supplementary Table 1). Human ZIKV-negative serum samples were collected from patients living in rural areas of Alfenas, before 2012, as there were no reports of ZIKV circulation in Brazil before 2013. The lack of reactivity against ZIKV of these samples was evaluated using an *in-house* ELISA, employing the domain III of the ZIKV envelope protein as antigen (supplementary Table 1). However, these samples were not systematically evaluated for prior exposure to other flaviviruses and therefore cannot be considered flavivirus-naive. PCR and IgG positive serum samples for *Orthoflavivirus denguei* (DENV) and *Alphavirus chikungunya* (CHIKV) were kindly provided by Prof. Danilo Bretas de Oliveira (Federal University of the Jequitinhonha and Mucuri Valleys). All experimental procedures were approved by the Research Ethics Committee of the Federal University of Alfenas (approval no. 6.802.332). In some experiments, commercial male human plasma (Sigma-Aldrich, USA), used for internal cut-off normalization, was also evaluated using the EDIII-based ELISA and did not show detectable anti-ZIKV reactivity under the analytical conditions employed. This material was used as a standardized analytical reference rather than as a serum derived from a biologically representative arbovirus-negative population. Serum panels were selected to enable controlled analytical assessment of antibody recognition patterns. Variations in the number of samples analyzed among peptides were related to limitations in serum availability and sample volume, as well as experimental optimization requirements.

**Table 1:** Synthetic peptides based on ZIKV structural proteins

ID	Sequence	Size (aa)	Protein
PEP 01	MMLELDPPF	9	Envelope
PEP 02	LNTKNGSISLMCLAL	15	Envelope
PEP 03	PSLGLINRWGSVGKK	15	Capsid
PEP 04	RRGADTSVGIVGLLL	15	Capsid
PEP 05	IQIMDLGHMCDATMS	15	prM

### Analytical evaluation of antibody class–specific seroreactivity

Briefly, 96-well microtiter plates were coated with one of the five peptides (200 ng/well) in carbonate buffer (pH 9.6, 0.1 M). After incubation (18–24 h) at 4 °C, wells were washed with PBS containing 0.05% Tween-20 (PBST). Subsequently, the plates were blocked with 200 µL/well of blocking buffer (5% dry milk, 1% BSA in PBST) for 2 h at 37 °C. After three washes, 100 µL of human serum (diluted 1:100) was added in duplicate (technical replicates) and incubated for 1 h at 37 °C. After three washes, the plates were incubated for 1 h at 37 °C with 100 µL/well of horseradish-peroxidase-labeled anti-human IgM, anti-human IgG or anti-mouse IgG (Sigma Aldrich, USA) diluted 1:5000 in PBST. After four additional washes, the plates were incubated for 15 min with 100 µL of 3,3’,5,5’-Tetramethylbenzidine in phosphate-citrate buffer (0.1 M citric acid, 0.1 M sodium phosphate dibasic, pH 4.5). After 15 min, the reaction was stopped using an acidic stop solution consisting of 30 µL per well of 2 M sulfuric acid. The resulting absorbance was measured at 450 nm (Abs 450 nm) using a microplate reader. The reactivity index (RI) was calculated as the ratio between the optical density (OD450) obtained for each sample and the predefined cut-off value (RI = sample OD/cut-off). The cut-off was defined as the mean optical density obtained from repeated measurements of the internal negative control (male human plasma, Sigma-Aldrich) plus two standard deviations. The cut-off definition was established for analytical comparison purposes and should not be interpreted as a definitive clinical diagnostic threshold. Samples with RI values > 1.1 were considered reactive for comparative analytical purposes. RI normalization was used to reduce inter-assay variability and enable comparative evaluation of peptide reactivity profiles across independent experiments, as previously applied in exploratory studies investigating ZIKV serological targets. The ELISA performance was evaluated using descriptive analytical parameters, including the proportion of reactive samples among previously characterized positive sera and the proportion of non-reactive samples among previously characterized negative sera under the experimental conditions employed. These parameters were used as exploratory indicators of analytical discriminatory behavior rather than measures of analytical detection limits or clinical diagnostic accuracy and were calculated as described by Mehra et al. [30]. Due to limitations in human serum availability and sample volume, independent experimental replicates were not performed.

### Statistical analysis

GraphPad Prism version 9.3.1 (GraphPad Software, San Diego, California, USA) was used to construct graphs and perform statistical analysis. ELISA assays were performed using technical duplicates, and the mean RI value obtained for each individual biological serum sample was used for statistical analysis and graphical representation. Data normality was assessed using the Shapiro–Wilk test. As the majority of datasets, particularly ZIKV-positive groups, did not follow a Gaussian distribution, comparisons between groups were performed using the non-parametric Mann–Whitney test (two-tailed). Receiver operating characteristic (ROC) curve analysis was used to evaluate peptide performance across a continuous range of threshold values, providing a threshold-independent assessment of analytical discriminatory capacity. For this purpose, reactivity index (RI) values obtained from indirect ELISA assays were used as continuous variables. ROC curves were constructed using reactivity index values to visualize the discriminatory behavior of each peptide across different threshold settings and the values were calculated with corresponding 95% confidence intervals (95% CI).

## Results

**Fig. 1:**
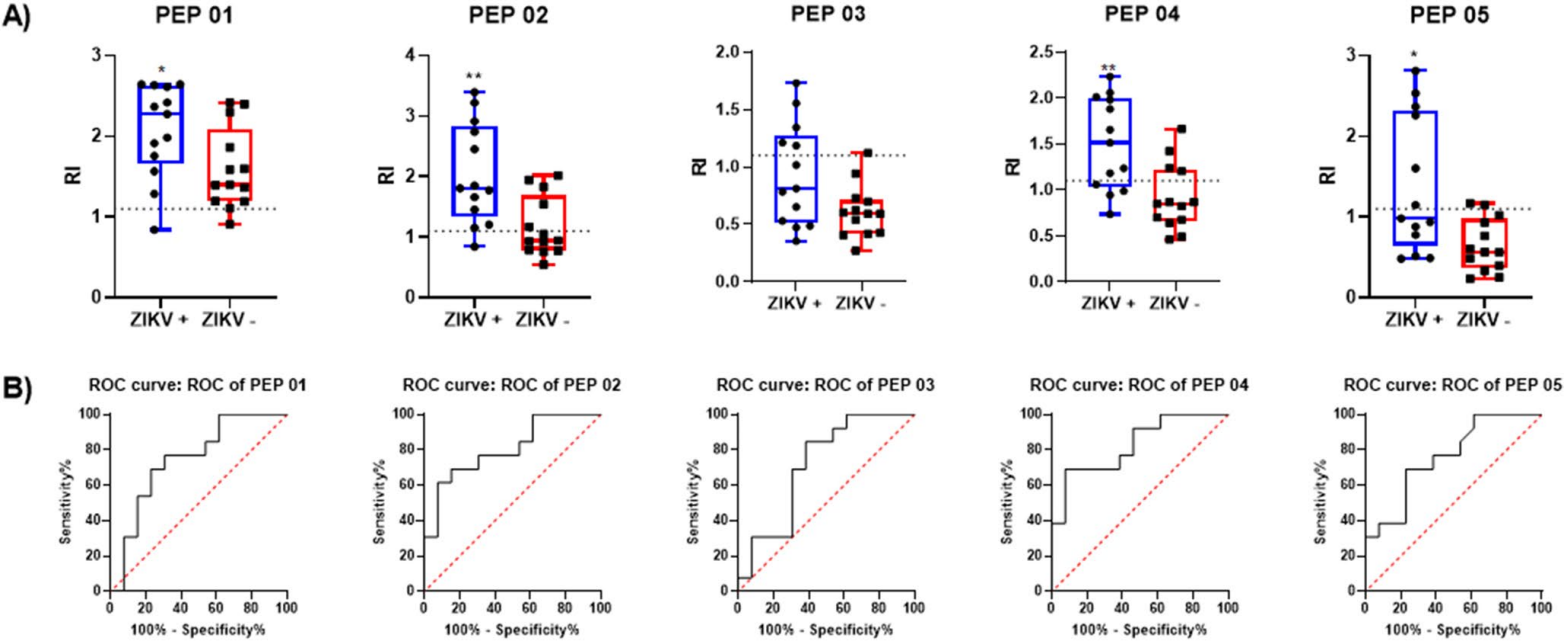
IgM reactivity to five synthetic ZIKV-derived peptides and corresponding ROC curve analysis. (**A**) Reactivity index (RI) obtained by indirect ELISA for five peptides using human serum ZIKV positive (*n* = 13) and negative (*n* = 13) samples. Each data point represents the mean RI value obtained from duplicate ELISA measurements of an individual biological serum sample. The dotted line indicates that values above this line are considered reactive (RI > 1.1). Differences were considered statistically significant when *p* < 0.05. P-values indicate differences in RI distributions between ZIKV-seropositive and ZIKV-seronegative groups and should not be interpreted as measures of diagnostic performance. (**B**) Receiver operating characteristic (ROC) curves for each peptide. Area under the curve (AUC) values are reported in the text

Five previously identified ZIKV-derived structural epitopes were selected for experimental evaluation of their antibody seroreactivity profiles. The IgM reactivity against the synthetic peptides were evaluated by indirect ELISA (Fig. 1A). Overall, all peptides tested showed a higher median value of reactivity index (RI) against sera from the ZIKV+ group compared with the ZIKV- group (supplementary Table 2). Most peptides showed statistically significant differences between ZIKV-positive and ZIKV-negative groups (PEP 01: *p* = 0.0387; PEP 02: *p* = 0.0072; PEP 04: *p* = 0.0040 and PEP 05: *p* = 0.0229), whereas PEP03 did not reach statistical significance under the conditions evaluated (*p* = 0.0622). However, these analyses were intended only to assess overall distributional differences and can not be used as an indicator of diagnostic performance. When the cut-off value of positivity of reactivity index (RI) was applied, it was observed that some peptides showed distinct analytical reactivity profiles between ZIKV-seropositive and ZIKV-seronegative samples. For PEP01, IgM reactivity was detected in 92.3% of ZIKV-positive samples (12/13), whereas only 1 of 13 (7.69%) ZIKV-negative samples remained below the reactivity threshold. For PEP 02, a similar pattern of reactivity was observed among positive samples (12/13, 92.3%), but among the ZIKV negative samples, 8 of 13 samples remained non-reactive. PEP 03 demonstrated reactivity in 5 of 13 positive samples (38.5%) and all ZIKV negative samples were classified as non-reactive. For PEP 04, reactivity observed for ZIKV-positive samples was 69.2% (9/13) and for ZIKV-negative sera, 9 of 13 (69.2%) were classified as negative samples. Finally, PEP 05 showed IgM reactivity in 46.2% of positive samples (6/13), whereas 11 of 13 (84.6%) ZIKV-negative samples were classified as non-reactive. Given that statistical differences in RI distributions alone do not establish analytical discriminatory utility, receiver operating characteristic (ROC) curve analysis was used as the principal comparative approach to evaluate peptide performance (Table 2; Fig. 1B). Among the peptides tested, PEP 02 and PEP 04 showed the highest discriminatory capacity, with AUC values above 0.8 (0.8047 for PEP 02 and 0.8225 for PEP 04), indicating comparatively favorable analytical discriminatory behavior under the experimental conditions employed. A second group of peptides presented moderate discriminatory capacity with AUC values below 0.8 (0.7396 for PEP 01, 0.7160 for PEP 03 and 0.7604 for PEP 05).

**Table 2:** Analytical reactivity parameters of synthetic ZIKV-derived peptides evaluated by indirect ELISA

	Sensitivity		Specificity		AUC	
	IgM (%)	IgG (%)	IgM (%)	IgG (%)	IgM (%)	IgG (%)
Pep 01	92.30	68.4	7.69	61.1	73.96 (0.5405 to 0.9388)	79.24 (0.6446 to 0.9402)
Pep 02	92.30	61.1	61.53	64.28	80.47 (0.6353 to 0.9742)	75 (0.5827 to 0.9173)
Pep 03	38.46	60	92.30	100	71.60 (0.5103 to 0.9217)	91.83 (0.8301 to 1.000)
Pep 04	69.23	60	69.23	93.3	82.25 (0.6609 to 0.9841)	87 (0.7550 to 0.9850)
Pep 05	46.15	70	84.61	80	76.4 (0.5760 to 0.9447)	86.38 (0.7533 to 0.9742)

*AUC *area under the curve

**Fig. 2:**
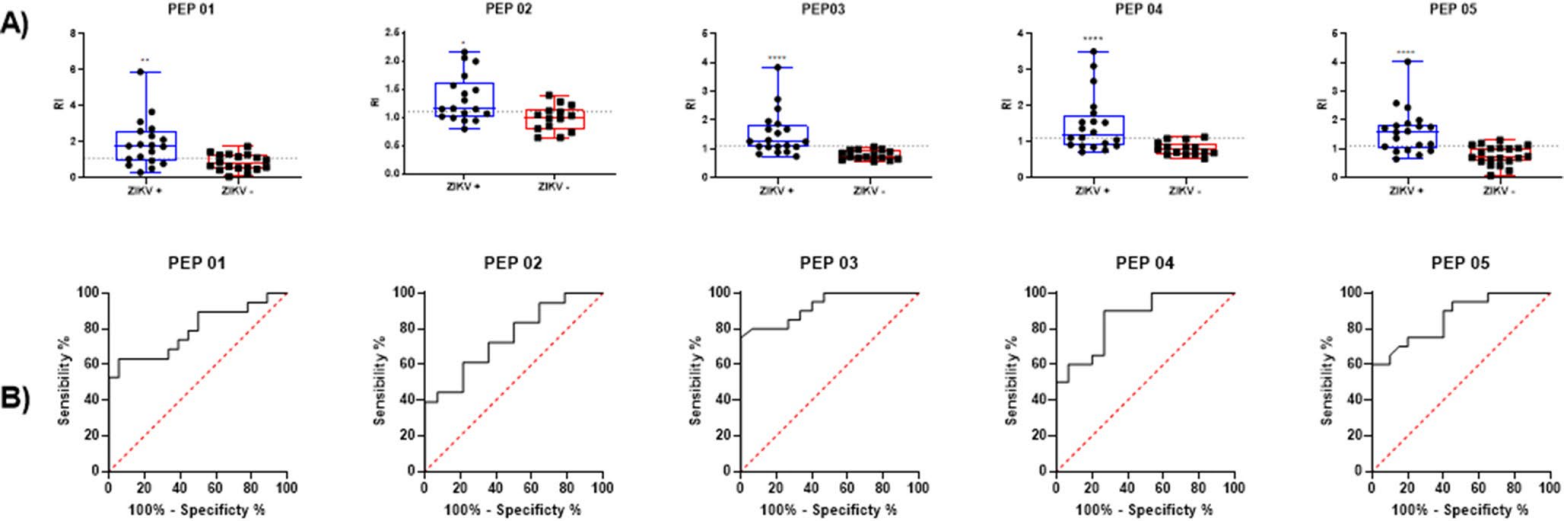
IgG reactivity to five synthetic ZIKV-derived peptides and corresponding ROC curve analysis. (**A**) Reactivity index (RI) obtained by indirect ELISA for five peptides using human serum ZIKV positive (*n* = 14–20) and negative (*n* = 15–20) samples. Each data point represents the mean RI value obtained from duplicate ELISA measurements of an individual biological serum sample. The dotted line indicates that values above this line are considered reactive (RI > 1.1). Differences were considered statistically significant when *P* < 0.05. P-values indicate differences in RI distributions between ZIKV-seropositive and ZIKV-seronegative groups and should not be interpreted as measures of diagnostic performance. (**B**) Receiver operating characteristic (ROC) curves for each peptide. Area under the curve (AUC) values are reported in the text

Next, we evaluated the reactivity of a panel of IgG ZIKV-positive samples against the five peptides, and due to limitations in serum availability, sample volume, and experimental optimization requirements, the number of samples analyzed varied among peptides (Fig. 2A). All peptides showed statistically significant differences in RI values between ZIKV-positive and ZIKV-negative groups (supplementary Table 3) (PEP 01: *p* = 0.0019; PEP 02: *p* = 0.0160; PEP 03: *p* < 0.0001; PEP 04: *p* < 0.0001 and PEP 05: *p* < 0.0001). However, when the positivity cut-off was applied (RI > 1.1), a differential performance was observed between the peptides. For PEP 01, IgG reactivity was detected in 68.4% of ZIKV-positive samples (13/19) and 61.1% (11/18) of negative samples remained non-reactive. For PEP 02, a similar pattern of reactivity was observed among positive samples 61.1% (11/18), but among the ZIKV-negative samples, 9 of 14 samples remained non-reactive (64.28%). PEP 03 demonstrated reactivity in 12 of 20 positive samples (60%) and all ZIKV negative samples (*n* = 15) were classified as non-reactive. For PEP 04, reactivity observed for ZIKV-positive samples was also 60% (12/20) and for ZIKV-negative sera, 1 of 15 (6.6%) were classified as positive samples. Finally, PEP 05 showed IgG reactivity in 70% of positive samples (14/20), whereas 16 of 20 (80%) ZIKV-negative samples were classified as non-reactive. According to the AUC values described by Hosmer et al. (2013), PEP 01 and PEP 02 demonstrated acceptable discriminatory power, with AUC values of 79.24% and 75%, respectively (Table 1; Fig. 2). PEP 04 (87%) and PEP 05 (86.38%) also showed comparatively favorable analytical discriminatory profiles, while PEP03 exhibited the most favorable analytical discriminatory profile under the experimental conditions employed, combining comparatively high apparent specificity, absence of detectable reactivity among the evaluated DENV-positive sera, and the highest AUC value among the peptides tested (91.83%). Although ROC curves were used as exploratory analytical descriptors rather than definitive diagnostic metrics, AUC values provided an additional threshold-independent parameter that supported comparative peptide prioritization (Table 2; Fig. 2A).

The analytical discriminatory profiles of the synthetic peptides for the detection of anti-ZIKV IgM and IgG antibodies were evaluated based on sensitivity, specificity values, and area under the ROC curve (AUC). AUC values are presented with corresponding 95% confidence intervals (95% CI) (Table 2). A distinct performance profile was observed among the peptides and between antibody classes. For IgM detection, PEP 01 and PEP 02 showed the highest proportion of reactive positive samples (92.30%), but this was accompanied by low specificity, particularly for PEP 01 (7.69%), indicating a high rate of false positives. PEP 04 showed similar values for sensitivity and specificity (both 69.23%), and PEP 05 exhibited moderate reactivity among positive samples (46.15%) with high specificity (84.61%). For IgG detection, PEP 03 demonstrated the most favorable profile, combining reactivity among positive samples (60%) with high apparent specificity (100%), whereas PEP 01 and PEP 02 showed moderate sensitivity (68.4% and 61.1%) and specificity (61.1% and 64.28%), while PEP 04 and PEP 05 presented intermediate sensitivity (60% and 70%) and good specificity (93,3% and 80% respectively).

**Fig. 3:**
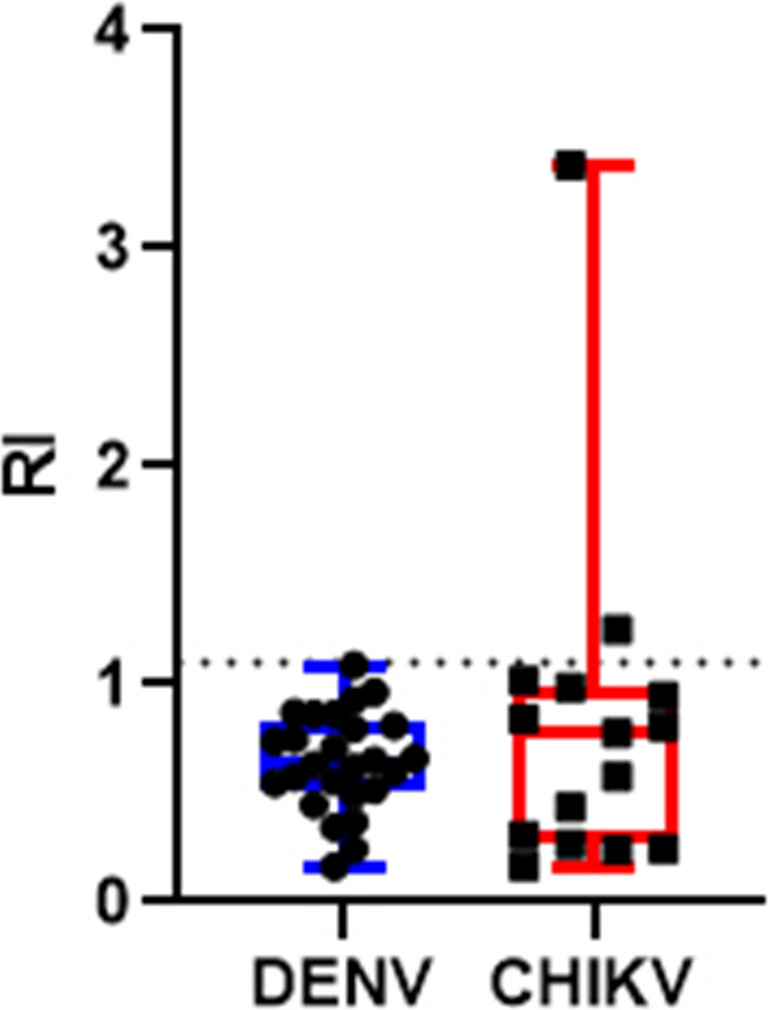
Cross-reactivity analysis of PEP 03 by indirect ELISA using DENV-positive and CHIKV-positive human serum samples. The reactivity index (RI) values obtained for individual positive sera are shown as individual data points. The dotted horizontal line represents the positivity threshold (RI > 1.1)

Since PEP 03 was the only peptide that demonstrated better analytical discriminatory power in IgG reactivity tests, it was evaluated for cross-reactivity with positive serum samples from another clinically relevant arbovirus in Brazil, including *Orthoflavivirus denguei (*DENV) and *Alphavirus chikungunya* (CHIKV) (Fig. 3 and supplementary Table 4). A total of 28 IgG-positive DENV were used, and among these, none of the DENV-positive samples evaluated exceeded the predefined analytical reactivity threshold. Additionally, 15 IgG-positive CHIKV serum samples were evaluated, and two CHIKV-positive samples exhibited detectable reactivity above the predefined analytical threshold.

## Discussion

The accurate identification of ZIKV infection remains a significant challenge due to the similarity of its clinical manifestations with other viral infections, particularly co-circulating arboviruses. This factor compromises clinical diagnosis and, consequently, patient management and epidemiological assessment [1]. In tropical and subtropical regions where ZIKV, *Orthoflavivirus denguei* (DENV) and *Alphavirus chikungunya* (CHIKV) share the same vector (*Aedes aegypti*), laboratory confirmation becomes essential for differential diagnosis [17]. However, in addition to clinical limitations, the high structural similarity among *Orthoflavivirus* species and the resulting cross-reactivity directly impact the reliability of available serological assays. This scenario is further aggravated by the phylogenetic diversity of ZIKV, which may influence viremia levels and the magnitude of the host immune response, thereby hindering laboratory detection of infection [18, 31–33].

In this context, reverse vaccinology and immunoinformatics may provide useful tools for preliminary antigen prioritization as alternatives to conventional antigen selection strategies that rely predominantly on E and NS1 proteins [20], acting as valuable tools for the rational selection of conserved and virus-specific epitopes, enabling the development of candidate antigens with reduced cross-reactivity [26, 34]. The present study was designed as an analytical proof-of-concept to evaluate ZIKV epitopes derived from structural proteins previously identified based on sequence and predicted accessibility, and not as a clinically validated diagnostic assay [27]. Our objective was to perform an initial comparative analytical evaluation of these epitopes and determine the antibody class-specific seroreactivity patterns and potentially favorable discriminatory profiles under controlled experimental conditions.

Evaluation of IgM reactivity (Fig. 1) in human sera revealed a pattern distinct from that observed for IgG (Fig. 2). Although all peptides exhibited significantly higher median reactivity index (RI) values in the ZIKV-positive group compared with the negative group, the application of a cut-off revealed relevant differences in antigenic performance among peptides. The differential performance observed among peptides may reflect their origin from structurally and immunologically distinct viral proteins. Peptides derived from the envelope protein showed higher sensitivity but lower specificity, consistent with the well-established immunodominance and cross-reactivity of this antigen in flavivirus infections. In contrast, the capsid-derived peptide PEP03 exhibited a more favorable profile, suggesting that less immunodominant but more virus-specific regions may provide improved discriminatory capacity for serological applications. These findings reinforce the concept that, for exploratory serological applications, antigen selection should prioritize specificity over immunodominance, particularly in settings where prior flavivirus exposure may drive cross-reactive antibody responses through immune imprinting mechanisms. For diagnostic applications, the most suitable epitope is not necessarily the most immunodominant, but the one that has the most analytically discriminatory under the experimental conditions employed.

In particular, PEP 01 and PEP 02 showed high proportions of reactive positive samples for IgM detection (92.3%), indicating that these epitopes are widely recognized by antibodies produced during the early stages of infection. However, this sensitivity was accompanied by a marked reduction in specificity, especially for PEP 01, which exhibited only 7.69% specificity, suggesting a high rate of false-positive results. This behavior is consistent with the intrinsic characteristics of early humoral responses, in which IgM antibodies present lower affinity and higher cross-reactivity, particularly in flavivirus infections, where structural homology promotes transient recognition of conserved epitopes. Accordingly, peptides showing favorable analytical performance in IgG assays may still exhibit limited discriminatory behavior during IgM detection, particularly when structurally conserved regions are recognized during early humoral responses [28, 29].

The extensive structural conservation among proteins of these viruses may favor nonspecific recognition of shared epitopes during the acute phase of the immune response [18]. In contrast, PEP 03 exhibited an opposite profile for IgM detection, with low reactivity among ZIKV-positive samples (38.46%) and high specificity (92.30%), suggesting that this epitope is recognized only by a subset of individuals during the early phase of infection but with a reduced likelihood of nonspecific reactivity. PEP 04 showed intermediate performance, with comparable proportions of reactive positive samples and specificity values (both 69.23%), whereas PEP 05 exhibited moderate reactivity among positive samples and high specificity. ROC curve analysis reinforced these observations, indicating that only PEP 02 and PEP 04 reached AUC values above 0.8 for IgM, reflecting good, but not excellent, analytical discriminatory capacity under the experimental conditions employed. Within the scope of this proof-of-concept study, the use of synthetic peptides for anti-ZIKV IgM detection appears conceptually feasible. However, this approach remains limited by intrinsic features of early humoral responses and by the high antigenic similarity among flaviviruses. Additional strategies, such as the combination of multiple epitopes into chimeric complexes or the use of alternative antigenic formats, may be required to improve discriminatory performance during this phase of infection [34].

Given the intrinsic limitations associated with IgM-mediated discrimination in flavivirus serology, the peptides were further evaluated in IgG assays, where affinity maturation mechanisms are expected to improve analytical specificity. Although several peptides showed statistically significant differences in RI distributions between groups, ROC analysis and threshold-based reactivity profiles revealed substantial variability in analytical discriminatory behavior. Notably, PEP03 showed the most favorable analytical discriminatory profile, combining favorable apparent sensitivity and specificity with the highest AUC value observed (91.83%) under the experimental conditions employed. The comparatively favorable analytical profile observed for IgG assays could be due to the effects of affinity maturation and clonal selection processes, which generally promote recognition of viral epitopes during more advanced stages of humoral responses [35]. The ability of PEP03 to maintain high apparent specificity suggests that this epitope may correspond to a region with favorable analytical specificity under the experimental conditions evaluated, capable of being recognized by a comparatively favorable IgG reactivity profile with limited detectable cross-reactivity. This hypothesis was further supported by cross-reactivity analyses performed using DENV and CHIKV positive sera (Fig. 3). Notably, none of the DENV samples tested were classified as reactive to PEP 03. Considering the well-established bidirectional cross-reactivity between antibodies against ZIKV and DENV [36], the absence of detectable reactivity among the DENV positive sera evaluated under the experimental conditions employed supports the analytical discriminatory potential of this peptide. Therefore, an additional validation using larger and immunologically diverse cohorts of flavivirus serum will be necessary to further assess its specificity. Minimal reactivity was observed among CHIKV-positive sera (2/15), despite the clinical and epidemiological relevance of CHIKV as a co-circulating arbovirus transmitted by the same vector. These observations are noteworthy given that serological cross-reactivity between ZIKV and DENV represents one of the major obstacles to the development of reliable diagnostic assays in endemic regions, but should be interpreted cautiously, particularly considering the limited sample size and the absence of fully characterized flavivirus-naive control cohorts. Nevertheless, it is important to note that flavivirus capsid proteins may contain structurally conserved regions capable of eliciting cross-reactive antibodies, and therefore, the absence of detectable reactivity observed in the present DENV panel should not be interpreted as definitive evidence of absolute specificity. Other peptides, such as PEP 04 and PEP 05, also showed satisfactory performance for IgG detection, with AUC values classified as very good, although with greater variability between sensitivity and specificity. PEP 01 and PEP 02 demonstrated acceptable performance but were mainly limited by specificity. These findings reinforce the importance of rational epitope selection and demonstrate that immunogenicity alone is not sufficient to define the suitability of a peptide for downstream applications. The comparative analysis of IgM and IgG responses indicates that the synthetic peptides evaluated here exhibit greater potential for IgG-oriented applications, including retrospective studies, serological surveillance, and late-stage differential assessment in endemic settings. The lower performance observed for IgM highlights limitations inherent to early humoral responses to flavivirus infection and suggests that additional optimization strategies would be required for applications targeting acute-phase detection. Importantly, beyond their relevance for exploratory serological applications, the rational antigen design approach employed in this study may also contribute to future vaccine and immunogen design strategies. The identification of structurally accessible and comparatively less cross-reactive epitopes may support the development of antigens with improved analytical specificity for serological platforms and may also assist in the selection of immunogenic regions for epitope-based vaccine design. In this context, capsid-derived epitopes such as PEP03 may represent underexplored antigenic targets with potential applicability in both differential serological approaches and future immunogen development studies.

Despite the promising results, this study has some limitations. Although ZIKV seronegativity of the control samples was experimentally confirmed using an EDIII-based ELISA, the reactivity of these sera to other flaviviruses cannot be excluded. This limitation is particularly relevant in endemic settings, where cross-reactive immune responses may influence serological outcomes. Therefore, the performance metrics reported in this study should be interpreted as analytical descriptors rather than definitive measures of diagnostic accuracy. Additionally, statistically significant differences in RI distributions between groups should not be interpreted as direct indicators of diagnostic applicability, since substantial overlap between reactivity profiles may still occur despite significant p-values. Cut-off values were established for analytical comparison purposes and should not be interpreted as clinical diagnostic thresholds. In addition, the relatively small sample size limits statistical power and reduces the precision and interpretability of ROC-derived parameters, including AUC estimates and confidence intervals. Therefore, ROC analysis should be interpreted as an exploratory comparative tool for analytical prioritization rather than a definitive assessment of diagnostic accuracy or clinically applicable true positive and false positive rates. The development and validation of serological tests for ZIKV are significantly hindered by the limited availability of biobanks containing well-characterized clinical samples [37]. Consequently, expanded validation using larger and rigorously characterized serum cohorts will be necessary to further define the translational applicability of these epitopes. Additionally, each peptide was evaluated individually because the primary objective of this study was to comparatively assess the analytical discriminatory behavior of each candidate epitope. This strategy enabled the identification of peptides with more favorable specificity profiles. Although combining epitopes may improve analytical performance and reduce the impact of viral genetic variability, the peptides investigated here were previously selected based on sequence conservation among circulating ZIKV strains. Future studies using chimeric or multiplexed antigenic platforms may further improve performance for broader serological applications [34]. Despite these limitations, PEP03 showed the most favorable IgG reactivity profile, with comparatively high specificity, absence of detectable reactivity among the evaluated DENV-positive sera, and minimal reactivity with CHIKV-positive samples. Although these viruses represent clinically relevant co-circulating arboviruses in the studied endemic context, additional evaluation against other flaviviruses will be important to more comprehensively define the analytical selectivity of these candidate epitopes. Notably, PEP03 is derived from the ZIKV capsid protein, a structural region that has been poorly explored as a serological antigen, since most available assays rely predominantly on envelope (E) and NS1 proteins [24, 38, 39]. Taken together, these findings provide preliminary analytical evidence that structurally derived ZIKV epitopes exhibit differential IgM and IgG recognition profiles and support their continued evaluation as candidate serological targets.

## Conclusion

This study provides a preliminary analytical evaluation of structurally derived ZIKV epitopes identified through immunoinformatics. Distinct IgM and IgG seroreactivity profiles were observed among the peptides evaluated, highlighting the influence of antigen origin and antibody class on analytical discriminatory behavior. Among the peptides tested, the capsid-derived peptide PEP03 exhibited the most favorable IgG analytical profile, combining high apparent specificity, favorable discriminatory capacity, and absence of detectable reactivity among the DENV-positive sera evaluated under the experimental conditions employed. The comparatively best performance of PEP03 supports the rationale in silico selection process, which prioritized conserved, structurally accessible, and less homologous regions among related flaviviruses. Although these findings should be interpreted as exploratory observations intended for early-stage antigen prioritization rather than validation of a clinically applicable diagnostic strategy, they support the continued evaluation of capsid-derived epitopes as candidate serological targets for ZIKV. Further studies using larger and well-characterized cohorts, including sera from additional flavivirus infections, will be necessary to further assess the translational applicability and analytical selectivity of these candidate epitopes.

## Supplementary Information

Below is the link to the electronic supplementary material.

Supplementary Material 1 (DOCX 2.27 MB)

Supplementary Material 2 (DOCX 18.2 KB)

Supplementary Material 3 (DOCX 21.6 KB)

Supplementary Material 4 (DOCX 2.27 MB)

## Data Availability

No datasets were generated or analysed during the current study.
