# Supplementary material for: Identification of a capsid-derived Zika virus epitope with high IgG discriminatory performance
Source: Arch Virol. 2026 Jun 12;171(7):208. doi: 10.1007/s00705-026-06677-3 (PMC13263302; doi:10.1007/s00705-026-06677-3)
Supplement: Supplementary file 1 — Supplementary Material 1 (DOCX 2.27 MB) [file 705_2026_6677_MOESM1_ESM.docx]

Supplementary table 1 – ZIKV positive and negative samples used in this study

| Sample ID | RT-qPCR ZIKV | ZIKV IgM/IgG |
| --- | --- | --- |
| VA 38 | NT | IgM |
| PO 27 | NT | IgM |
| HP 123 | Negative | IgM |
| HP 80 | Positive | IgM |
| HP 44 | Negative | IgM |
| HP 142 | Positive | IgM |
| 30971 | Negative | IgM |
| 33246 | Negative | IgM |
| 31567 | Negative | IgM |
| 39032 | Negative | IgM |
| 242 | Negative | IgM |
| 29374 | Negative | IgM |
| 33060 | Negative | IgM |
| 409 | Positive | IgM |
| 368 | NT | IgG/IgM |
| 388 | NT | IgG/IgM |
| 252 | NT | IgG |
| 297 | NT | IgG |
| 311 | NT | IgG/IgM |
| 600 | NT | IgG |
| 578 | NT | IgG |
| 346 | NT | IgG |
| 527 | NT | IgG |
| 99 | Positive | IgG |
| 30 | Positive | IgG/IgM |
| 29 | Negative | NT |
| 28 | Positive | IgG |
| 27 | Positive | - |
| 25 | Positive | - |
| 26 | Negative | NT |
| 19 | Positive | IgG |
| 20 | Negative | NT |
| 21 | Positive | IgG/IgM |
| 26714 | Negative | Negative |
| 26823 | Negative | Negative |
| 26686 | Negative | Negative |
| 26922 | Positive | Negative |
| 27288 | Negative | Negative |
| 27353 | Negative | Negative |
| 27354 | Negative | Negative |
| 27336 | Negative | Negative |
| 27380 | Negative | Negative |
| 26818 | Negative | Negative |
| 27283 | Negative | Negative |
| 39283 | Negative | Negative |
| 39286 | Negative | Negative |
| 211 | NT | Negative |
| 207 | NT | Negative |
| 206 | NT | Negative |
| 205 | NT | Negative |
| 204 | NT | Negative |
| 203 | NT | Negative |
| 199 | NT | Negative |
| 197 | NT | Negative |
| 194 | NT | Negative |
| 192 | NT | Negative |
| 191 | NT | Negative |
| 190 | NT | Negative |
| 189 | NT | Negative |
| 188 | NT | Negative |
| 187 | NT | Negative |
| 185 | NT | Negative |
| 184 | NT | Negative |
| 183 | NT | Negative |
| 182 | NT | Negative |
| 181 | NT | Negative |
| 180 | NT | Negative |
| 213 | NT | Negative |
| 210 | NT | Negative |
| 208 | NT | Negative |
| 200 | NT | Negative |
| 195 | NT | Negative |
