# Supplementary material for: Identification of a capsid-derived Zika virus epitope with high IgG discriminatory performance
Source: Arch Virol. 2026 Jun 12;171(7):208. doi: 10.1007/s00705-026-06677-3 (PMC13263302; doi:10.1007/s00705-026-06677-3)
Supplement: Supplementary file 2 — Supplementary Material 2 (DOCX 18.2 KB) [file 705_2026_6677_MOESM2_ESM.docx]

|  |  |  | Reactivity Index (IgM) | | | | |
| --- | --- | --- | --- | --- | --- | --- | --- |
| Positive Sample | RT-qPCR ZIKV | ZIKV IgM | Pep 01 | Pep 02 | Pep 03 | Pep 04 | Pep 05 |
| VA 38 | NT | Positive | 2,643065 | 1,663297 | 0,473468 | 0,736291 | 0,938329 |
| PO 27 | NT | Positive | 2,36545 | 1,852616 | 1,017497 | 1,235745 | 0,880036 |
| HP 123 | Negative | Positive | 1,565519 | 0,845171 | 0,487785 | 1,182577 | 0,490789 |
| HP 80 | Positive | NT | 1,913253 | 1,809794 | 0,812975 | 1,654641 | 1,148936 |
| HP 44 | Negative | Positive | 1,984803 | 1,775987 | 0,784342 | 1,514472 | 0,983459 |
| HP 142 | Positive | Negative | 2,634479 | 2,916405 | 1,185205 | 2,057426 | 2,365567 |
| 30971 | Negative | Positive | 2,644496 | 3,396462 | 1,729233 | 1,980092 | 2,262144 |
| 33246 | Negative | Positive | 0,841431 | 1,153941 | 0,530734 | 0,945739 | 0,483268 |
| 31567 | Negative | Positive | 1,757273 | 1,455949 | 0,355868 | 0,98924 | 0,515235 |
| 39032 | Negative | Positive | 2,276728 | 2,456632 | 1,343709 | 1,880201 | 1,603997 |
| 242 | Negative | Positive | 2,613014 | 3,220667 | 1,554367 | 2,233041 | 2,532924 |
| 29374 | Negative | Positive | 2,419829 | 2,742863 | 1,211792 | 2,010703 | 2,811226 |
| 33060 | Negative | Positive | 1,285042 | 1,208032 | 0,652425 | 1,058519 | 0,778493 |

|  |  |  | Reactivity Index (IgM) | | | | |
| --- | --- | --- | --- | --- | --- | --- | --- |
| Negative Sample | RT-qPCR ZIKV | ZIKV IgM | Pep 01 | Pep 02 | Pep 03 | Pep 04 | Pep 05 |
| 26714 | Negative | Negative | 2,299624 | 1,945021 | 0,602317 | 0,866794 | 0,767211 |
| 26823 | Negative | Negative | 1,588415 | 0,933069 | 0,408021 | 0,493009 | 0,564126 |
| 26686 | Negative | Negative | 1,863168 | 1,169717 | 0,698442 | 1,422637 | 0,934568 |
| 26922 | Positive | Negative | 1,39666 | 0,775304 | 0,417225 | 0,866794 | 0,566006 |
| 27288 | Negative | Negative | 2,416967 | 1,816555 | 0,941823 | 1,238967 | 1,148936 |
| 27353 | Negative | Negative | 1,190596 | 0,759527 | 0,427451 | 0,675068 | 0,336595 |
| 27354 | Negative | Negative | 1,598432 | 1,048013 | 0,728098 | 0,741125 | 0,398649 |
| 27336 | Negative | Negative | 1,399522 | 0,547671 | 0,54096 | 0,462397 | 0,235052 |
| 27380 | Negative | Negative | 1,363747 | 1,543847 | 0,593114 | 1,205133 | 1,03423 |
| 26818 | Negative | Negative | 0,91155 | 0,788827 | 0,593114 | 0,823293 | 0,250096 |
| 27283 | Negative | Negative | 2,398363 | 2,019396 | 0,273037 | 1,662697 | 1,199707 |
| 39283 | Negative | Negative | 1,107597 | 0,944338 | 1,122825 | 0,849071 | 0,607375 |
| 39286 | Negative | Negative | 1,29649 | 0,917293 | 0,624814 | 0,639623 | 0,485148 |
