# Supplementary material for: Identification of a capsid-derived Zika virus epitope with high IgG discriminatory performance
Source: Arch Virol. 2026 Jun 12;171(7):208. doi: 10.1007/s00705-026-06677-3 (PMC13263302; doi:10.1007/s00705-026-06677-3)
Supplement: Supplementary file 3 — Supplementary Material 3 (DOCX 21.6 KB) [file 705_2026_6677_MOESM3_ESM.docx]

|  |  |  | Reactivity Index (IgG) | | | | |
| --- | --- | --- | --- | --- | --- | --- | --- |
| Positive Sample | RT-qPCR ZIKV | ZIKV IgM/IgG | Pep 01 | Pep 02 | Pep 03 | Pep 04 | Pep 05 |
| 409 | Positive | - | 1,7384 | 1,3064 | 1,2677 | 1,1186 | 1,7289 |
| 368 | NT | IgG/IgM | 3,1164 | 1,9982 | 2,7369 | 2,6869 | 2,5919 |
| 388 | NT | IgG/IgM | 1,8573 | 1,0667 | 1,1231 | 1,3734 | 1,0870 |
| 252 | NT | IgG | - | - | 1,0992 | 0,7733 | 0,6497 |
| 297 | NT | IgG | 2,7305 | 1,5689 | 1,9664 | 1,8025 | 1,7850 |
| 311 | NT | IgG/IgM | 1,4628 | 1,1479 | 1,6881 | 1,5488 | 1,7652 |
| 600 | NT | IgG | 0,3053 | 0,7978 | 0,8329 | 0,8528 | 0,9434 |
| 578 | NT | IgG | 0,9493 | 1,0206 | 1,2605 | 0,9650 | 0,9649 |
| 346 | NT | IgG | 0,5142 | 1,7403 | 0,7445 | 0,8898 | 0,7900 |
| 527 | NT | IgG | 5,8984 | 2,1639 | 3,8382 | 3,5147 | 4,0390 |
| 99 | Positive | IgG | 3,6733 | 2,0612 | 2,4084 | 3,1128 | 2,4450 |
| 30 | Positive | IgG/IgM | 0,7057 | 0,9953 | 1,0921 | 1,1077 | 1,1448 |
| 29 | Negative | NT | 2,1213 | 0,9446 | 0,9021 | 0,8822 | 1,5853 |
| 28 | Positive | IgG | 1,1698 | 0,9440 | 1,0730 | 0,9149 | 1,1695 |
| 27 | Positive | - | 2,3331 | 1,1622 | 1,5543 | 1,5368 | 1,6200 |
| 25 | Positive | - | 1,0451 | 1,0804 | 0,8842 | 0,7177 | 1,3758 |
| 26 | Negative | NT | 0,7840 | - | 1,0825 | 1,0554 | 0,9154 |
| 19 | Positive | IgG | 1,7790 | 1,4896 | 1,3107 | 1,2667 | 1,8097 |
| 20 | Negative | NT | 2,5913 | 1,4240 | 1,6881 | 1,9746 | 2,0045 |
| 21 | Positive | IgG/IgM | 1,8167 | 1,1531 | 1,8757 | 1,5651 | 1,8873 |

|  |  |  |  |  |  |  |  |
| --- | --- | --- | --- | --- | --- | --- | --- |
|  |  |  | Reactivity Index (IgG) | | | | |
| Negative Sample | RT-qPCR ZIKV | ZIKV IgG | Pep 01 | Pep 02 | Pep 03 | Pep 04 | Pep 05 |
| 211 | NT | Negative | 1,318 | 0,869925 | 0,927 | 0,976 | 1,14 |
| 207 | NT | Negative | 0,633 | 0,643878 | NT | NT | 0,572 |
| 206 | NT | Negative | 1,741 | 0,84784 | 0,977 | 1,135 | 1,194 |
| 205 | NT | Negative | 1,123 | 0,737415 | 0,68 | 0,57 | 0,876 |
| 204 | NT | Negative | 0,572 | 1,033615 | 0,74 | 0,748 | 0,638 |
| 203 | NT | Negative | 0,99 | 0,979701 | 0,863 | 0,86 | 0,686 |
| 199 | NT | Negative | 0,436 | 0,639981 | 0,588 | 0,64 | 0,08 |
| 197 | NT | Negative | 0,54 | 1,045956 | 0,971 | 0,846 | 0,584 |
| 194 | NT | Negative | 1,431 | 0,981 | 0,98 | 1,052 | 1,009 |
| 192 | NT | Negative | 0,662 | NT | 0,685 | 0,716 | 0,712 |
| 191 | NT | Negative | 0,476 | NT | 0,57 | 0,693 | 0,678 |
| 190 | NT | Negative | 1,176 | NT | NT | NT | 1,313 |
| 189 | NT | Negative | 1,266 | NT | NT | NT | 1,016 |
| 188 | NT | Negative | NT | NT | 0,809 | 0,852 | 0,247 |
| 187 | NT | Negative | 1,283 | NT | NT | NT | 1,145 |
| 185 | NT | Negative | NT | NT | 1,073 | 1,024 | 1,033 |
| 184 | NT | Negative | 0,073 | NT | 0,762 | 0,647 | 0,419 |
| 183 | NT | Negative | 0,218 | NT | 0,725 | 0,707 | 0,437 |
| 182 | NT | Negative | NT | NT | NT | NT | NT |
| 181 | NT | Negative | 0,587 | NT | 0,647 | 0,652 | 0,749 |
| 180 | NT | Negative | 0,88 | NT | NT | NT | 1,028 |
| 213 | NT | Negative | NT | 1,120656 | NT | NT | NT |
| 210 | NT | Negative | NT | 1,39672 | NT | NT | NT |
| 208 | NT | Negative | NT | 1,221338 | NT | NT | NT |
| 200 | NT | Negative | NT | 1,103118 | NT | NT | NT |
| 195 | NT | Negative | NT | 1,279799 | NT | NT | NT |
