# Supplementary material for: Identification of a capsid-derived Zika virus epitope with high IgG discriminatory performance
Source: Arch Virol. 2026 Jun 12;171(7):208. doi: 10.1007/s00705-026-06677-3 (PMC13263302; doi:10.1007/s00705-026-06677-3)
Supplement: Supplementary file 4 — Supplementary Material 4 (DOCX 2.27 MB) [file 705_2026_6677_MOESM4_ESM.docx]

Supplementary Table 4: *Orthoflavivirus denguei* positive samples used in this study

|  |  |  | Reactivity Index (IgG) |
| --- | --- | --- | --- |
| DENV Sample | RT-qPCR DENV | DENV IgM/IgG | Pep 03 |
| 28647 | Positive | IgG | 0,709064695 |
| 28674 | Positive | Negative | 0,165472524 |
| 28718 | Positive | Negative | 0,518417718 |
| 28724 | Positive | IgM/IgG | 0,966026397 |
| 28727 | Positive | IgM/IgG | 0,651675744 |
| 28737 | Positive | IgG | 0,810831083 |
| 28739 | Positive | IgM/IgG | 1,089101497 |
| 28750 | Positive | IgM/IgG | 0,602221748 |
| 28753 | Positive | IgM/IgG | 0,748912572 |
| 35602 | Positive | IgM/IgG | 0,738607748 |
| 35613 | Positive | Negative | 0,66079009 |
| 35625 | Positive | Negative | 0,927178103 |
| 35715 | Positive | IgM/IgG | 0,866495464 |
| 35917 | Positive | IgG | 0,631624182 |
| 35734 | Positive | Negative | 0,368879585 |
| 35735 | Positive | IgM/IgG | 0,870608622 |
| 35742 | Positive | IgM/IgG | 0,797819569 |
| 35761 | Positive | IgM/IgG | 0,865140014 |
| 35764 | Positive | IgM/IgG | 0,549657925 |
| 35776 | Positive | IgM/IgG | 0,448960116 |
| 35837 | Positive | Negative | 0,555377963 |
| 35841 | Positive | IgM/IgG | 0,344679425 |
| 35848 | Positive | IgM/IgG | 0,61998252 |
| 35852 | Positive | IgM/IgG | 0,573983801 |
| 35870 | Positive | IgM/IgG | 0,49365184 |
| 35940 | Positive | IgM/IgG | 0,246244488 |

Supplementary Table 3: *Alphavirus chikungunya* positive samples used in this study

|  |  |  | Reactivity Index (IgG) |
| --- | --- | --- | --- |
| CHIKV Sample | RT-qPCR CHIKV | CHIKV IgM/IgG | Pep 03 |
| 26737 | Positive | IgM | 0,779500569 |
| 291223 | Positive | IgM | 0,840183208 |
| 26788 | Positive | IgM/IgG | 0,1661011 |
| 30871 | Positive | IgM | 0,307593465 |
| 31001 | Positive | Negative | 0,24567877 |
| 26791 | Positive | Negative | 0,986580818 |
| 26794 | Positive | Negative | 0,578572402 |
| 31005 | Positive | IgM | 0,436891464 |
| 26843 | Positive | Negative | 1,018135313 |
| 31145 | Positive | IgM | 0,944906257 |
| 26844 | Positive | IgM | 0,240901595 |
| 31254 | Positive | Negative | 0,268433206 |
| 33359 | Positive | Negative | 0,807248202 |
